# The challenge of intracellular temperature

**DOI:** 10.1007/s12551-020-00683-8

**Published:** 2020-03-14

**Authors:** Madoka Suzuki, Taras Plakhotnik

**Affiliations:** 1 Institute for Protein Research, Osaka University, 3-2 Yamadaoka, Suita, Osaka 565-0871 Japan; 2 School of Mathematics and Physics, The University of Queensland, St Lucia, Brisbane, QLD 4072 Australia

**Keywords:** Fluctuation, Fluorescence, Heat, Heat conductivity, Kapitza resistance, Lipid bilayer, Microscopy, Nanothermometer, Statistical mechanics, Thermodynamics

## Abstract

This short review begins with a brief introductory summary of luminescence nanothermometry. Current applications of luminescence nanothermometry are introduced in biological contexts. Then, theoretical bases of the “temperature” that luminescence nanothermometry determines are discussed. This argument is followed by the 10^5^ gap issue between simple calculation and the measurements reported in literatures. The gap issue is challenged by recent literatures reporting single-cell thermometry using non-luminescent probes, as well as a report that determines the thermal conductivity of a single lipid bilayer using luminescence nanothermometry. In the end, we argue if we can be optimistic about the solution of the 10^5^ gap issue.

## Current situation of luminescence nanothermometry in biology

Maintaining homeostasis of our body temperature upon cold exposure requires the ways of releasing heat in our bodies. These processes are called shivering thermogenesis (if they take place in skeletal muscle) and non-shivering thermogenesis (Tseng et al. 2010; Bal et al. 2012). Many studies have been dedicated to identify molecular cues and signal transduction cascades that govern the thermogenesis. However, it is still unclear when, where, and how much the heat is released at the subcellular resolution, especially, in a quantitative manner. This is a situation where the methods using the optical microscopy to quantify temperature at the subcellular resolution have been developed extensively. In this article, we call these optical methods as “luminescence nanothermometry.”

Luminescence nanothermometry relies on luminescent temperature probes that are usually fluorescent proteins and luminescent molecules or nanoparticles. Currently, there is a huge variety of luminescent temperature probes. The variety in materials and thermometry methods that also include non-luminescent probes have been well summarized in excellent review articles (Brites et al. 2012; Jaque and Vetrone 2012; Quintanilla and Liz-Marzánab 2018). Schematic energy diagrams of probes can also be found (Quintanilla and Liz-Marzánab 2018). In the current short paper, we only focus on the materials that have been applied for intracellular thermometry (Okabe et al. 2018) (Table 1). One of the best advantages of using these probes in cell biology is that they are compatible with other luminescent probes and optical devices measuring intracellular parameters. Combinations with fluorescent Ca^2+^ (Suzuki et al. 2007; Takei et al. 2014; Arai et al. 2014; Itoh et al. 2016) or pH (Hou et al. 2017) probes have identified spatial and temporal correlations between thermogenesis and these intracellular parameters at the single cellular level to identify unresolved mechanisms of thermogenesis. Chrétien et al. have employed an oxygen-sensitive optode device to correlate changes in mitochondrial temperature with oxygen consumption. They demonstrated that the mitochondrial temperature is possibly increased up to 323 K in both human embryonic kidney 293 cells and primary skin fibroblasts due to full activation of respiration (Chrétien et al. 2018).

**Table 1:** Summary of luminescent materials that have been applied for intracellular thermometry

Material	Size, nm	Resolution			Analyzed parameter of luminescence				
		Spatial, nm	Thermal, K	Temporal, sec	Intensity	Spectrum (band shape)	Lifetime	Anisotropy	Others
Organic molecule	10^0^	10^2^*	10^−1^	10^−2^	✓				
Polymer^†^	10^0^~10^2^	10^2^*	10^−1^	10^−2^	✓	✓	✓	✓	
Particle	10^0^~10^2^	10^2^*	10^−2^	10^−2^	✓	✓	✓		ODMR^#^

✓Parameters that have been applied for intracellular luminescence thermometry

^†^Including proteins and synthetic polymers

^*^Diffraction-limited, 200–300 nm

^#^Optically detected magnetic resonance microscopy

The resolution is described as the order of magnitude. The resolution is not examined in cells in some cases. The thermal and temporal resolutions are not necessarily achieved at the same time and sometimes only one of the two values is reported. References (Brites et al. 2012; Jaque and Vetrone 2012; Okabe et al. 2018; Quintanilla and Liz-Marzánab 2018)

Herein, we begin with asking if the “temperature” can be considered at the scale where luminescence nanothermometry functions. We next introduce the paradox in luminescence nanothermometry that we call “the 10^5^ gap issue,” a contradiction between calculation and measurement. We discuss if there could be a solution of the paradox by referring to recent studies using luminescence and non-luminescence nanothermometry.

## The conceptual validity of temperature and its fluctuations on a nanoscale

Pressure, volume, entropy, temperature, and internal energy are macroscopic properties in classical thermodynamics and as such, they do not fluctuate. In statistical mechanics, a small system in contact with a thermal bath continuously exchanges its energy with the bath and this results in fluctuations. While fluctuations of mechanical quantities—volume, energy, and force (pressure)—are easy to visualize because these quantities are microscopic in their nature (can be assigned to a single atom), the concept of fluctuations is less obvious for temperature because temperature is a parameter deduced from statistical distributions. Some people even think that temperature may not fluctuate.

One conceptual approach to temperature fluctuations is to consider temperature as a thermodynamical state function of mechanical quantities. For example, temperature and internal energy of the small system at constant volume, *T*_s_ and *U*_s_ , respectively, are related to each other so that $\Delta  {U}_{\mathrm{s}}={V}_{\mathrm{s}}\mathcal{C}\mathcal{\Delta  }{T}_{\mathrm{s}}$, where $\mathcal{C}$ is the volumetric heat capacity (we do not distinguish in this review the heat capacities at constant volume and constant pressure because their values are very close in solids and liquids), *V*_s_ is the volume of a small system, and *∆* stands for a change. Using this equality, uncertainty in the internal energy can be linked to the uncertainty in temperature.

A different conceptual approach to temperature fluctuations has been proposed by Phillies (Phillies 1984) where a small system is considered as a thermometer and its energy is used to determine the temperature of a thermal bath which is in contact with the thermometer. In this case, the uncertainty in the readings of the thermometer is treated as uncertainty of the inferred bath temperature.

Interestingly, the two approaches outlined above predict a similar value of fluctuations. This can be shown as follows. The conditional probability distribution *P*(*U*_s_| *T*_b_), that is, the probability of *U*_s_ given *T*_b_, the temperature of a thermal bath, describes statistical uncertainty in *U*_s_, while probability *P*(*T*_b_| *U*_s_) describes uncertainty in *T*_b_. The relation between *P*(*T*_b_| *U*_s_) and *P*(*U*_s_| *T*_b_) is set by Bayes’ theorem (see a textbook on statistics, for example, Sivia 1996) and in a simplified form reads *P*(*T*_b_| *U*_*s*_) ∝ *P*(*U*_s_| *T*_b_). The state equation ${\overline{U}}_{\mathrm{s}}\left({T}_{\mathrm{b}},{\overline{V}}_{\mathrm{s}}\right)$ linearized within a small range of *T*_b_ reads ${\overline{U}}_{\mathrm{s}}={U}_0+{\overline{V}}_{\mathrm{s}}\mathcal{C}{T}_{\mathrm{b}}$ (the bars above the corresponding symbols indicate mean values). The probability of ${\overline{U}}_{\mathrm{s}}$ depends on the deviation of the energy from the mean value, that is, *P*(*U*_s_| *T*_b_) = *P*(*x*), where $x={U}_{\mathrm{s}}-{\overline{V}}_{\mathrm{s}}\mathcal{C}{T}_{\mathrm{b}}-{U}_0$. According to the error propagation rule, ${\sigma}_x={\sigma}_{U_{\mathrm{s}}}$ (*T*_b_ is fixed) and ${\sigma}_x={\sigma}_{T_{\mathrm{b}}}\mathcal{C}{\overline{V}}_{\mathrm{s}}$ (*U*_s_ is fixed), and therefore, ${\sigma}_{U_{\mathrm{s}}}={\overline{V}}_{\mathrm{s}}\mathcal{C}{\sigma}_{T_{\mathrm{b}}}$. According to the state equation ${\sigma}_{U_{\mathrm{s}}}={\overline{V}}_{\mathrm{s}}\mathcal{C}{\sigma}_{T_s}$, it follows that ${\sigma}_{T_{\mathrm{s}}}={\sigma}_{T_{\mathrm{b}}}$.

Despite the conceptual difference of the two approaches to temperature fluctuations, in both cases, it is assumed that the small system would be in thermodynamic equilibrium with a thermal bath (otherwise the thermodynamic temperature cannot be defined), but it is not in exact thermal equilibrium with the bath it is actually in contact. An interesting discussion of conceptual problems related to temperature at a quantum level can be found in Ghonge and Vural (2018). But this topic is outside of this short review.

In the following text, we drop subscript “s” for briefness when referring to the system. The expression for the standard deviation of temperature fluctuations *σ*_T_ in a classical textbook (Landau and Lifshitz 1980) reads as follows:

1 $$ {\sigma}_{\mathrm{T}}=T\sqrt{\frac{k_{\mathrm{B}}}{\overline{V}\mathcal{C}}} $$

where *k*_B_ is Boltzmann’s constant. Choi et al. have already discussed the formula in a recent paper (Choi et al. 2019) in relation to the temperature measurements on nanoscale (see Eq. (3) there). To grasp an intuitive understanding of the origin of these fluctuations, we may consider Debye’s model for the heat capacity. In a high temperature limit, this model predicts that $\overline{V}\mathcal{C}=3N{k}_{\mathrm{B}}$ where *N* is the number of atoms in the small system. In this case,

2 $$ {\sigma}_{\mathrm{T}}=\frac{\overline{T}}{\sqrt{3N}} $$

This expression has a simple physical meaning as 3*N* is the number of classical degrees of freedom if the atoms making the nanocrystal are considered as point masses.

Remarkably, simple estimates based on Eq. (1) are in a good agreement with complex molecular dynamics simulations. For example, calculated fluctuations of temperature of a single amino acid residue reported by Takayanagi and Nagaoka (2011) are about 70 K. According to Eq. (1), this value equals *σ*_T_ in a spherical volume of water ($\mathcal{C}=4.2\times {10}^6\ \mathrm{J}{\mathrm{K}}^{-1}{\mathrm{m}}^{-3}$) with a radius of *a* = 0.25 nm. These fluctuations look quite large because even 1° of temperature change may be a significant stress for a biological system. However, we should consider a time factor to assess the significance of these fluctuations. This is done in the next paragraph. We conclude this paragraph by noting that a small nanocrystal of diamond (*a* = 50 nm and $\mathcal{C}=1.9\times {10}^6\ \mathrm{J}{\mathrm{K}}^{-1}{\mathrm{m}}^{-3}$) has temperature fluctuations of about 0.03 K.

When one measures temperature of a single small system for an extended period of time, the readings will change randomly with a characteristic correlation time *τ*. The *τ* is equal to the time it takes to exchange the energy between the bath and the small system, and the standard deviation is defined by Eq. (1). But if the actual measurement time *t*_m_ is much longer than *τ*, the result will be the average of *t*_m_/*τ* times of statistically uncorrelated energy exchanging events, and ${\overline{\sigma}}_{\mathrm{T}}$, the standard deviation of the average, will be $\sqrt{t_{\mathrm{m}}/\tau }$ times smaller than *σ*_*T*_. A similar approach is applicable if time *t*_m_ is a time required for a significant change in the system. For example, it can be the time of a chemical reaction. The value of ${\overline{\sigma}}_{\mathrm{T}}={\sigma}_{\mathrm{T}}\sqrt{\tau /{t}_{\mathrm{m}}}$ can be much smaller than *σ*_T_.

The characteristic time *τ* can be determined by considering heat exchange between the small system and the bath. For simplicity, we consider a small system which has a shape of a sphere with radius *a*. In such a case, an estimate for the relaxation time reads (Philip 1964) as follows:

3 $$ \tau \approx {\left(\frac{{\mathcal{C}}_{\mathrm{i}}}{{\mathcal{C}}_{\mathrm{o}}}\right)}^{2/3}\frac{a^2}{\alpha_{\mathrm{o}}} $$

where the subscripts “o” and “i” referred to the regions outside and inside of the small system, respectively. The factor in front of *a*^2^/*α*_o_ is approximately 1 for most common pairs of materials. For example, it is 0.6 for diamond-water and 0.7 for gold-water pairs. A spherical region in water bath (*a* = 1.5 nm and *α*_o_ = 1.43 × 10^−7^*m*^2^*s*^−1^) has the relaxation time of about 15 ps. This number is very close to the results of molecular dynamics simulations of the temperature relaxation times (10–20 ps) in protein molecules surrounded by water (Lervik et al. 2010). The relaxation time is about 12 ns for a typical diamond nanocrystal (*a* = 50 nm) in water.

If a fluorescent nanodiamond is used as a luminescent temperature probe, the precision of the temperature measurement is typically inversely proportional to the square root of the measurement time. One of the reasons is a fluctuation of the number of detected photons. It is therefore convenient to characterize such a thermometer by a noise floor $\upeta \equiv {\sigma}_{\mathrm{m}}\sqrt{t_{\mathrm{m}}}$, where *σ*_m_ is the measurement error. One can estimate the smallest possible value of η defined by thermodynamic fluctuations of the thermometer as ${\upeta}_{\mathrm{min}}={\overline{\sigma}}_{\mathrm{T}}\sqrt{\tau }$. It is about 3 μKs^1/2^ for diamond (*a* = 50 nm) in water. For comparison, in a recent paper (Choi et al. 2019), experimentally achieved record-low noise floor in all-optical measurements was about 10 mK s^1/2^. Therefore, intrinsic fluctuation of temperature is not a limiting factor even in the current state-of-the-art luminescence nanothermometry.

We have considered two conceptual approaches to the temperature fluctuations. First, temperature is considered as a state function of mechanical variables subject to mechanical fluctuations. Second, a small system is considered as a thermometer, and its fluctuations define inaccuracy of the inferred bath temperature. The two concepts are in quantitative agreement in relation to the temperature uncertainties. In most cases, the concept of temperature is valid even for a region of only 10-nm across. Fluctuations at this size are on the order of 1 K (the value is inversely proportional to the square root of the volume of the system) but they are very fast and have a characteristic time on the order of 0.1 ns (proportional to the square of the size). If response of a physical system is relatively slow, the fluctuations are averaged out and the effective value of the fluctuations is reduced to a much smaller number (inversely proportional to the square root of the response time). The thermodynamics limited value of the noise floor for a 50-nm thermometer is several μK s^1/2^, which is more than three orders of magnitude smaller than the current record experimentally achieved.

## Five orders of magnitude disagreement between calculations and measurements

There is a paradox in intracellular nanothermometry. The amplitude of the temperature increase can be calculated using parameters determined in experiments. However, the calculated values are usually orders of magnitude smaller than the values obtained experimentally. This gap between calculation and measurement is called as “the 10^5^ gap issue”. For example, Yang et al. measured local temperature increase of about 1 K using their Q-dot based nanothermometry in NIH3T3 cell line upon Ca^2+^ shock. When the authors calculated the heat released from the local heat source, they found that a power of about 1 μW or larger is required to achieve 1-K increase (Yang et al. 2011). This value seems even three orders of magnitude larger than the values determined in stimulated brown adipocytes that are known as thermogenic cells (Nedergaard et al. 1977; Clark et al. 1986; Johannessen et al. 2002; Kriszt et al. 2017). Suzuki’s group calculated that the whole cell temperature can be increased only by 10 μK when the sarco-/endoplasmic reticulum Ca^2+^-ATPase, Serca, is solely responsible for the measured temperature changes in HeLa cells upon Ca^2+^ shock (Takei et al. 2014; Arai et al. 2014). Furthermore, the presence of a stable temperature variation over 1 K among cellular compartments has been demonstrated in unstimulated cells using different kinds of luminescence thermometry (Okabe et al. 2012; Kiyonaka et al. 2013; Hayashi et al. 2015; Tanimoto et al. 2016; Nakano et al. 2017; Uchiyama et al. 2015; Uchiyama et al. 2018). These measurements require additional considerations on the cellular heat power and make the gap even wider. Baffou et al. summarized the gap issue in their critique, and it was followed by a discussion among researchers working in the field (Baffou et al. 2014; Kiyonaka et al. 2015; Suzuki et al. 2015; Baffou et al. 2015; Uchiyama et al. 2017).

## Measurable temperature increase using non-luminescent probes

Should the temperature increase be undetectable in individual cells? It may not be the case when the temperature was measured using non-luminescent thermal probes. One example can be found in a report where Sato et al. (2014) described the temperature increase for about 0.2 K using bimetal microcantilevers for stimulated brown adipocytes. The authors also claimed the difficulty to explain the gap between their results and calculations.

Two more recent studies using microfabricated thermocouples also report a temperature increase in individual cells, one in relatively longer (Yang et al. 2017) and the other on shorter time scales (Rajagopal et al. 2019). The approach by Yang et al. was to build their high-performance micro-thermocouple arrays, over which adherent cells were cultured, within a double-stabilized tent (Yang et al. 2017). Their thermally stabilized system enabled measurements in individual adherent human hepatoblastoma cells in unstimulated conditions for days. The authors detected frequent fluctuations of about 60 mK during each measurement for over 30 h, as well as a continuous elevation up to 285 mK in one of the detection areas, while other areas remained stable in a measurement for about 40 h long. Rajagopal et al. (2019) employed a biocompatible microscale thermocouple probe to detect temperature increase in the vicinity of mitochondria in *Aplysia californica* neurons. By stimulating targeted cells using proton uncoupler, the authors observed rapid temperature increases of about 7.5 K that can be fit by a biexponential curve. The short-term component had the amplitude of about 4.8 K with a time constant of about 1 s. As the time constant matches the temporal response of proton currents, it was suggested that the short-term component corresponds to the heat release caused by the induced proton uncoupling.

Considering the variety of probes and methods using luminescence and non-luminescence nanothermometry, it may be unnecessary to conclude that the temperature increase is unmeasurable in individual cells. However, how could it be possible? The reason may be uncovered in the parameters that have been used to calculate the amplitude of temperature increase and that, at the same time, have experimentally undetermined yet.

## Can better understanding of thermal conductivity in a cell resolve the controversy?

One of the possible parameters causing the discrepancy between calculations and measurements is the thermal conductivity in a living cell. In calculations, the thermal conductivity in cells has been hypothesized as the same value as the one in water. This assumption may be inappropriate as studies using computational methods report the thermal conductivity of proteins (Yu and Leitner 2003; Leitner 2008; Lervik et al. 2010) and of lipid bilayers (Potdar and Sammalkorpi 2015; Wang et al. 2016; Youssefian et al. 2018) as two to six times smaller than the value of water. However, experimental data have been missing until Bastos et al. (2019) successfully determined the value of a single lipid bilayer very recently.

Bastos et al. covered the surface of upconversion nanoparticles by lipid bilayer. The radiation of the near-infrared light (980 nm) is mainly absorbed by the nanoparticles and the water thus causes a transient heating in the suspension of these nanoparticles. Temperature changes of the suspension were measured using the luminescence of the nanoparticles and the thermocouple that was immersed in the suspension. The authors observed larger temperature increase using nanoparticles covered by lipid bilayers than the value determined by the thermocouple. However, the gap was absent when the temperature increase was determined using nanoparticles without lipid bilayers. By analyzing the data using a steady-state temperature model based on the lumped resistance, the thermal conductivity of the lipid bilayer and its temperature dependence were successfully determined. The thermal conductivity of a single lipid bilayer was about 0.2 W m^−1^ K^−1^ at 300 K, which is three times smaller than that of water.

The experimental results by Bastos et al. strongly support the possibility that the local thermal conductivity in cells is significantly smaller than that of water. It is probably possible to hypothesize heterogeneous values in a cell when we consider the site-specific architecture and components of biomolecules including proteins and lipids that form intracellular organelle and cellular morphology. The complexity of a cell interior breaks down the continuum approximation for the medium in the heat conductivity equation. This hypothesis is, in principle, valid, but to justify it, quantitative estimation of the expected effects is required. Thus, we next discuss boundary resistance problem.

The boundary resistance, also called Kapitza resistance, is a well-known but not well-understood effect when the temperature shows “discontinuity” on an interface between two different materials. The temperature discontinuity at the interface, *∆T*_i_, can be estimated as follows:

$$ \Delta  {T}_{\mathrm{i}}=\frac{\dot{Q}}{A}{R}_{\mathrm{t}} $$

where $\dot{Q}$ is the power of heat release, *A* is the interface area, and *R*_t_ is the thermal resistance on the boundary.

Numerical modeling which considers a single protein molecule in water estimates *R*_*t*_ at the water-protein interface to be in the range of (0.4–1.0) × 10^−8^ K m^2^ W^−1^. Thermal resistance of a lipid bilayer has also been investigated by numerical simulations (Ge et al. 2006; Nakano et al. 2010). The modeling results in *R*_t_ = 1.7 × 10^−8^ K m^2^ W^−1^. A similar value was obtained by molecular dynamic modeling of water-octane interface (Patel et al. 2005). Thermal resistance of a hydrophobic layer (octadecylsilane, C18) has been determined experimentally and has a value of *R*_t_ ~ 2 × 10^−8^ K m^2^ W^−1^ (Ge et al. 2006). Note that these values are about 2 times larger than the temperature resistance between water and a protein.

For better understanding of the significance of these boundary effects, the equation above can be rewritten as follows:

$$ -{\upkappa}_{\mathrm{eff}}\frac{\Delta  {T}_{\mathrm{i}}}{\delta r}=\frac{\dot{Q}}{A} $$

where *κ*_eff_ *= δr/R*_t_ is the effective thermal conductivity and *δr* is the thickness of the interface which, unlike in the case of mathematical models, consists of atoms and molecules. For example, *δr* is ~ 3 nm for a lipid bilayer (Andersen and Koeppe II 2007). The value of *κ*_eff_ = 0.1 W m^−1^ K^−1^ is estimated for the case *δr* = 2 nm and *R*_t_ ~ 2 × 10^−8^K m^2^ W^−1^. Then, one can see that *κ*_eff_ is close to the values of proteins (Yu and Leitner 2003; Leitner 2008; Lervik et al. 2010) and thus that all the interfaces can be simply replaced with a protein layer of approximately the same thickness. According to this modeling, the interior of the cell can be treated as a medium with the thermal conductivity of about 0.1 Wm^−1^ K^−1^ on average. To conclude, the gap still remains although it gets narrower. Perhaps this gives some ground for an optimistic future to close the gap.

Before closing this section, we would comment on other complexed factors that may contribute significantly to intracellular thermogenesis. An aspect of complexity is that the heat flow and the corresponding local temperature change may significantly affect chemical and physical processes in cells. Temperature shifts the equilibrium in chemical reactions and affects flows driven by electrochemical gradients (Kondepudi and Prigogine 1998). Temperature gradients have been shown to generate directional motions of particles (Duhr and Braun 2006), as well as to induce accumulation of nucleotides (Baaske et al. 2007) and lipids (Budin et al. 2009). A stable pH gradient can also be formed due to thermally separated buffer molecules of different charge states in the solution (Keil et al. 2017). These heat-induced processes may in return affect the processes of intracellular thermogenesis locally. These complexities are poorly understood and studied experimentally in cells but can be essential factors in theoretical considerations.

## Conclusion

In this short review, we have discussed mainly two issues that are present in luminescence nanothermometry, which are briefly summarized in Table 2. The first one was a general issue in nanothermometry, i.e., the conceptual validity of the “temperature” at the small scale. It was shown that the concept of temperature is valid even on a scale of 10 nm. While the fluctuations of the temperature can reach 1 K at 10-nm scale, the characteristic correlation time is very short, on the order of 0.1 ns. Therefore, the fluctuations can be averaged out in most methods and by relatively slow physical and chemical processes. The second issue discussed was the “10^5^ gap.” The temperature increase that is calculated from parameters in literatures can be up to five orders of magnitude smaller than that determined experimentally in luminescence nanothermometry. This discussion was extended by consideration of three studies using non-luminescence methods that have reported measurable temperature increases in individual cells. Then, we focused on the intracellular value of the thermal conductivity as it is one of the parameters not yet investigated experimentally although recent measurements show that the thermal conductivity of a lipid bilayer is about three times smaller than that of water. Considering the boundary heat resistance problem, the average thermal conductivity was calculated in cells as up to six times smaller than that in water. The gap still remains, and the “10^5^ gap issue” is still an open question. One has to consider arguments and approach the gap from both sides. The theoretical estimates can be a bit relaxed when the complexity of processes in cells is understood better. However, the experimental results should also be critically considered, and new methods of temperature measurement more accurate and less susceptible to artifacts should be developed.

**Table 2:** Current issues in luminescence nanothermometry

Category	Description	Comments
Validity of temperature in luminescence nanothermometry	• In aqueous conditions and luminescent temperature probes, the concept of temperature is valid even on a scale of 10 nm. • Temperature fluctuations can be on the order of 1 K at 10-nm scale with a characteristic correlation time on the order of 0.1 ns.	• The fluctuations can be averaged out in most of the current luminescence thermometry and be reduced.
The 10^5^ gap issue	• Amplitude of temperature increase calculated using parameters well identified is unable to explain values determined in measurements.	• Non-luminescent thermal probes also report temperature increases in individual cells. • Thermal conductivity of a single lipid bilayer experimentally determined was about three times smaller than that of water. • Calculations including boundary heat resistance problem expect the average thermal conductivity in cells up to six times smaller than that in water. • This issue is still an open question.

It is clear that luminescence nanothermometry and its application to cell biology involve a variety of research disciplines. Methods to be developed could have potential usages for diagnosis and medical purposes. However, as we argued here, this research field holds important questions that need to be solved. The issues can also involve fundamental questions, not only in biology but also in physics in thermodynamics and statistical mechanics; the definition of the temperature; and the way to consider the heat at the nanoscale in solution and in individual biological molecules.
